# Rare primary bone tuberculosis associated with ruxolitinib therapy: A case report and literature review

**DOI:** 10.1097/MD.0000000000050140

**Published:** 2026-08-07

**Authors:** Liang-Yan Jin, Ping Yu, Fan Huo, Ya-Hui Cui, Bao-Liang Wu, Ting Li

**Affiliations:** a Department of Pharmacy, Hangzhou Xixi Hospital, Hangzhou Sixth People’s Hospital, Hangzhou Xixi Hospital Affiliated to Zhejiang Chinese Medical University, Hangzhou, 310023, Zhejiang, China; b Department of Neurosurgery, Hangzhou Xixi Hospital, Hangzhou Sixth People’s Hospital, Hangzhou Xixi Hospital Affiliated to Zhejiang Chinese Medical University, Hangzhou, 310023, Zhejiang, China; c Department of Radiology, Hangzhou Xixi Hospital, Hangzhou Sixth People’s Hospital, Hangzhou Xixi Hospital Affiliated to Zhejiang Chinese Medical University, Hangzhou, 310023, Zhejiang, China.

**Keywords:** adverse reaction, Janus kinase inhibitor, primary bone tuberculosis, ruxolitinib

## Abstract

**Rationale:**

Ruxolitinib, a Janus kinase (JAK) inhibitor, may increase the risk of tuberculosis infection by suppressing cellular immunity. Tuberculosis associated with ruxolitinib has generally been reported as disseminated or multisystem disease. However, no previous cases of primary bone tuberculosis secondary to ruxolitinib have been reported.

**Patient concerns:**

A 68-year-old female patient had been taking ruxolitinib for over 2 years for primary thrombocythaemia and developed recurrent high fever without other localizing symptoms.

**Diagnoses:**

Imaging studies revealed multiple areas of bone destruction with increased metabolic activity throughout the skeleton (sacroiliac joints, humeral heads), necessitating differential diagnosis from malignant tumors. Following exclusion of hematological malignancies via bone marrow aspiration, Xpert MTB/RIF (X-pert) testing of the bone lesion tissue confirmed *Mycobacterium tuberculosis* infection. Concurrently, bronchial alveolar lavage fluid analysis ruled out active pulmonary tuberculosis, leading to a definitive diagnosis of isolated primary bone tuberculosis confined to the skeletal system.

**Interventions:**

Standard four-drug antituberculosis therapy was administered once daily: isoniazid 200 mg, rifampicin 450 mg, pyrazinamide 1125 mg, and ethambutol 625 mg, while ruxolitinib was continued for the underlying myeloproliferative disorder.

**Outcomes:**

Follow-up MRI examinations of the right humeral head and sacroiliac joints at 4 and 5 months post-tuberculosis treatment revealed a marked reduction in lesion size and decreased edema at both sites, consistent with an effective therapeutic response..

**Lessons:**

This case shows that ruxolitinib-associated tuberculosis can present as an isolated skeletal infection. It may occur without any pulmonary or systemic involvement. Latent tuberculosis screening should be performed before initiating ruxolitinib. Vigilance for extrapulmonary tuberculosis should be maintained throughout therapy. Unexplained bone lesions in JAK-inhibitor users warrant multidisciplinary evaluation. Empirical fluoroquinolone use before diagnosis may transiently mask the disease and delay recognition. Finally, CYP3A4-mediated interactions should be anticipated when rifampicin-based therapy is combined with ruxolitinib.

## 1. Introduction

The oral Janus kinase (JAK) inhibitor ruxolitinib was initially approved by the US Food and Drug Administration in 2011 for the treatment of patients with intermediate or high-risk myelofibrosis (MF), including primary myelofibrosis (PMF), post-polycythemia vera MF, and post-essential thrombocythemia MF.^[1,2]^ Ruxolitinib primarily functions by inhibiting the abnormal activation of cytokines through the blockade of the JAK-STAT signaling pathway, thereby serving as a treatment for myeloproliferative disorders.^[3]^ However, the JAK-STAT signaling pathway plays a pivotal role in the activation of immune cells and the secretion of cytokines, which are crucial for the body’s immune defence against intracellular pathogens.^[4]^ Interferon-γ (IFN-γ) signals through JAK1 and JAK2 to activate macrophages and drive granuloma formation, which is central to containing *Mycobacterium tuberculosis* (MTB).^[5]^ By inhibiting JAK1 and JAK2, ruxolitinib blunts this IFN-γ-mediated response, impairs T-helper-1 (Th1) cell function, and lowers key cytokines such as IFN-γand TNF-α.^[6–8]^ The result is weakened cellular immunity against intracellular pathogens like MTB and a greater risk of opportunistic infections, including tuberculosis.^[9,10]^ This may be associated with a reduction in cellular immune function caused by the medication.^[11]^ In the general population, latent tuberculosis infection may remain asymptomatic for extended periods. When the body’s immune function is suppressed, latent MTB may reactivate, thereby triggering overt infection.

Bone tuberculosis is a secondary infectious disease caused by MTB invading the bone or joints. It most commonly arises secondary to pulmonary tuberculosis, with a minority of cases arising secondary to gastrointestinal tuberculosis or lymph node tuberculosis. Primary bone tuberculosis refers to the form that directly affects the bones without any other site having a clearly identified primary tuberculosis focus. It is extremely rare in clinical practice. The incidence of bone tuberculosis is <5% of all tuberculosis cases.^[12]^ The sacroiliac joint is a deep joint and is a relatively rare site for tuberculosis to occur. It is reported that its incidence accounts for approximately 5% to 10% of all cases of bone tuberculosis.^[13]^ The type of tuberculosis infection secondary to ruxolitinib typically presents as disseminated tuberculosis involving multiple sites. The infection may affect bones and joints, leading to the formation of bone tuberculosis.^[14]^ In recent years, with the widespread use of immunosuppressants and biologics, the incidence of bone tuberculosis among immunocompromised individuals has shown an upward trend, presenting new challenges for clinical diagnosis and treatment. Osteoarticular tuberculosis often has an insidious onset and a low bacterial load, and it can mimic tumors or inflammatory disease, so early diagnosis is difficult. In immunocompromised patients, including those on JAK inhibitors, the clinical picture is often atypical and blunted, which makes diagnosis even harder. Although the risk of tuberculosis with JAK inhibitors is increasingly recognized, isolated skeletal tuberculosis without lung or systemic involvement has not been reported with ruxolitinib.

In this report, the patient had been taking ruxolitinib long-term for thrombocytosis when recurrent high fevers developed. This ultimately led to a diagnosis of rare isolated bone tuberculosis, which did not involve the lungs or blood. To date, no relevant case reports have been documented. The emergence of this rare case further underscores the importance of closely monitoring patients for infections when using immunosuppressive drugs such as ruxolitinib. Particular vigilance is warranted regarding tuberculosis infection, which also provides practical case references for clinical investigations into the association between immunosuppressive agents and infectious diseases. Analysis of this case study contributes to a deeper understanding of the mechanisms underlying secondary bone tuberculosis associated with ruxolitinib, its clinical characteristics, and key diagnostic and therapeutic considerations. This report aims to raise clinical awareness of this rare adverse event and to highlight the need for pretreatment screening and further investigation.

## 2. Case report

A 68-year-old female patient weighing 47.5 kilograms presented with a history of over 2 years of primary thrombocythaemia, with a positive JAK2 gene mutation in exon 12 (68%). Long-term oral administration of ruxolitinib 20 mg twice daily has provided adequate control of platelet counts. Additionally, with a history of osteoporosis, calcium carbonate D3 tablets are administered; with a history of iron deficiency anemia and folate deficiency anemia, treatment comprises ferrous polysaccharide complex and folic acid tablets.

The patient developed recurrent fever approximately 1 month ago, with the highest recorded temperature exceeding 39°C, and no other significant symptoms. During an outpatient consultation at the hospital, a urine culture indicated the presence of Escherichia coli. A chest CT scan revealed multiple nodules and infectious lesions in both lungs, accompanied by a small amount of pleural effusion. Pelvic Magnetic Resonance Imaging (MRI) demonstrated bone resorption and destruction beneath the left sacroiliac joint surface with bone marrow edema, accompanied by abnormal soft tissue signals in the surrounding area; minimal bone marrow edema is noted in the right sacroiliac joint and bilateral ischial tuberosities. Preliminary consideration is for urinary tract infection and pulmonary infection.The patient was administered piperacillin-tazobactam and cefotiam for anti-infective therapy. The body temperature has not fully returned to normal, which is suspected to be related to inflammation of the left sacroiliac joint.

Seven days prior, the patient was transferred to another hospital. Positron Emission Tomography/Computed Tomography (PET-CT) revealed mixed bone destruction with increased metabolic activity in multiple skeletal sites throughout the body, including the right humeral head and both iliac bones. The histopathological examination of the soft tissue biopsy from the right humeral head revealed granulomatous nodules with necrosis within proliferative fibrous tissue, accompanied by focal multinucleated cell infiltration. Bone marrow aspiration and flow cytometry revealed no evidence of haematological malignancy. Following empirical administration of levofloxacin injection for infection control, the patient’s temperature returned to normal. However, persistent fatigue prompted referral to our hospital for further diagnostic clarification.

Upon admission, inflammatory markers showed a slight elevation, with neutrophil count at 8.09*10^9^ cells/L (87.6%) and C-reactive protein at 21.12 mg/L. Considering a presumed inflammatory/rheumatic sacroiliitis, the initial treatment regimen comprises oral administration of doxycycline enteric-coated capsules 0.1 g twice daily and prednisolone tablets 5 mg three times daily. Continue with the original treatment regimen of ruxolitinib and supportive care such as correction of anemia. Of note, baseline screening for latent tuberculosis infection had not been performed before ruxolitinib was started, as the patient had no history of tuberculosis, no known contact, and no respiratory or constitutional symptoms at that time.

On hospital day 3, the T-cell SPOT Test (Xiamen Innodx Biotech Co., Ltd., Xiamen, China) returned a positive result (19.09). MRI of the right upper arm demonstrates a long T1/long T2 signal in the right humeral head and neck, accompanied by surrounding tissue thickening and edema. Bilateral sacroiliac joint MRI demonstrates patchy and nodular hyperintense signal on T1-weighted and T2-weighted sequences beneath both sacroiliac joint surfaces, with high signal intensity on fat-suppressed sequences. The lesion is most prominent around the left sacroiliac joint, where a soft tissue mass measuring approximately 33 mm × 32 mm is visible, findings highly suggestive of a chronic granulomatous lesion. Medication regimen unchanged.

On day 9, a needle biopsy was performed on the skeletal lesion of the sacroiliac joint, a normally sterile deep skeletal site; owing to the sclerotic, difficult-to-sample nature of the lesion, an adequate specimen was obtained only after 5 biopsy attempts. Specimens were submitted for metagenomic next-generation sequencing (mNGS), Xpert MTB/RIF (Cepheid AB, Solna, Sweden), histopathological examination, and tissue culture. Pelvic computed tomography (CT) further confirmed diffuse bone destruction beneath both sacroiliac joint surfaces and within the left ilium, with localized soft tissue mass formation at the left sacroiliac joint and hypertrophic osteophytes at the lumbosacral joints.

On day 10, the X-pert bone tissue test returned a positive result for Mycobacterium tuberculosis complex DNA (at an extremely low level), with no mutations detected in the rifampicin resistance gene. Taken together with the positive T-SPOT.TB, the granulomatous nodules with necrosis identified on the earlier right humeral-head soft-tissue biopsy performed at the referring hospital (in which caseous necrosis was not explicitly documented), and the characteristic imaging findings, this led to a definitive diagnosis of bone tuberculosis. Doxycycline and prednisolone tablets were discontinued, and preparations were made for elective antituberculosis therapy.

**Figure 1.**
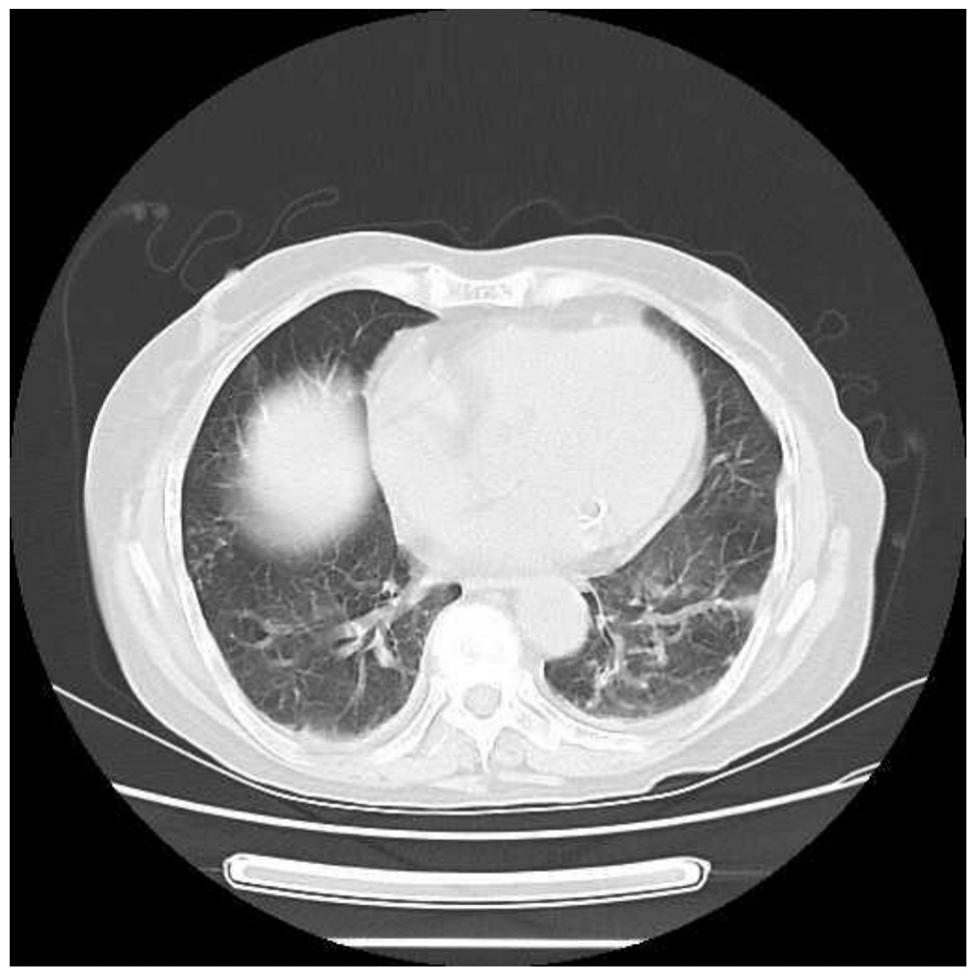
On admission, chest CT showed multiple tiny nodules and scattered inflammatory opacities in both lungs.

On day 12, the mNGS test for bone tissue returned negative, and the bone-tissue mycobacterial culture submitted on day 9 subsequently showed no growth. A bone-marrow biopsy revealed mature adipose tissue with focal spindle-cell stromal proliferation, scattered hematopoietic cells, and focally increased reticulin fibers; immunohistochemistry was positive for CD117 (occasional cells), CD45 (partial), Ki-67 (occasional cells), and MPO (few cells), and negative for CD34, with a negative iron stain. To rule out pulmonary tuberculosis, bronchoscopy was performed. The bronchoalveolar lavage fluid (BALF) Xpert MTB/RIF test and acid-fast bacillus culture were both negative. The remaining BALF work-up was similarly unremarkable: the smear showed only a few Gram-positive cocci, while brush culture, routine and Legionella cultures, and Pneumocystis smear were all negative; the Aspergillus galactomannan index was 0.17 (negative). The initial chest CT demonstrated multiple pulmonary nodules and scattered inflammatory opacities in both lungs but lacked the typical imaging features of tuberculosis, such as tree-in-bud signs, cavitation, or disseminated lesions (Fig. 1). Taken together, these findings clearly establish that the pulmonary lesions are nontuberculous in nature, and active pulmonary tuberculosis is ruled out.

On day 14, antituberculosis therapy was started with isoniazid 200 mg, rifampicin 450 mg, pyrazinamide 1125 mg, and ethambutol 625 mg once daily.

**Figure 2.**
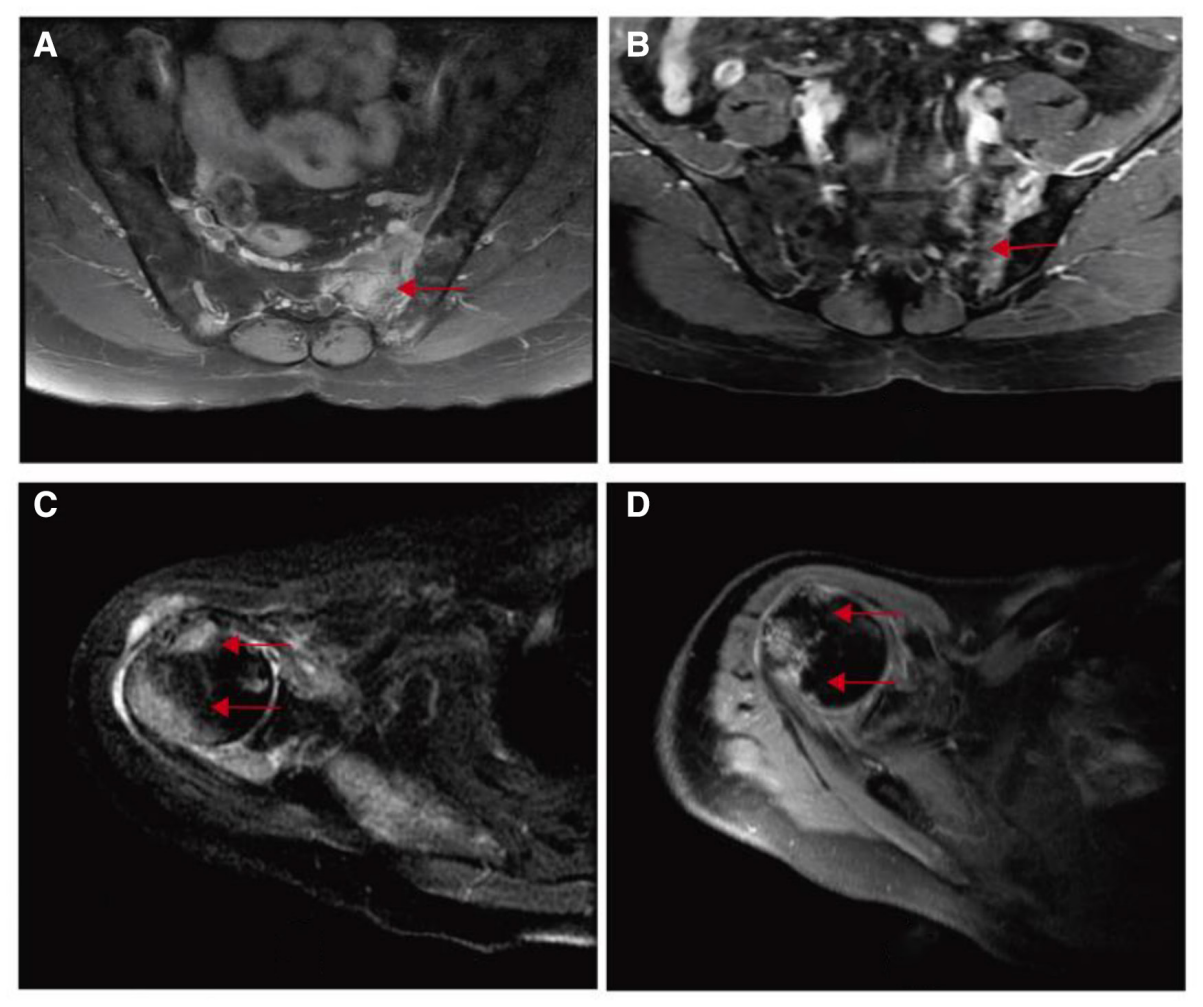
(A) On admission, pelvic MRI shows diffuse bony destruction beneath both sacroiliac joint surfaces, in the left iliac bone, and in the lumbosacral vertebrae, accompanied by localized soft-tissue mass formation at the left sacroiliac joint. (B) After 5 months of antituberculosis treatment, repeat pelvic MRI shows improvement of the bilateral sacroiliac joint surfaces and iliac bone compared with before. (C) On admission, MRI of the right upper arm demonstrates a long T1/long T2 signal in the right humeral head and neck, accompanied by surrounding tissue thickening and edema. (D) After 4 months of antituberculosis treatment, MRI of the right humeral head revealed abnormal bone signal intensity with surrounding tissue thickening and edema, showing improvement compared with previous findings.

**Figure 3.**
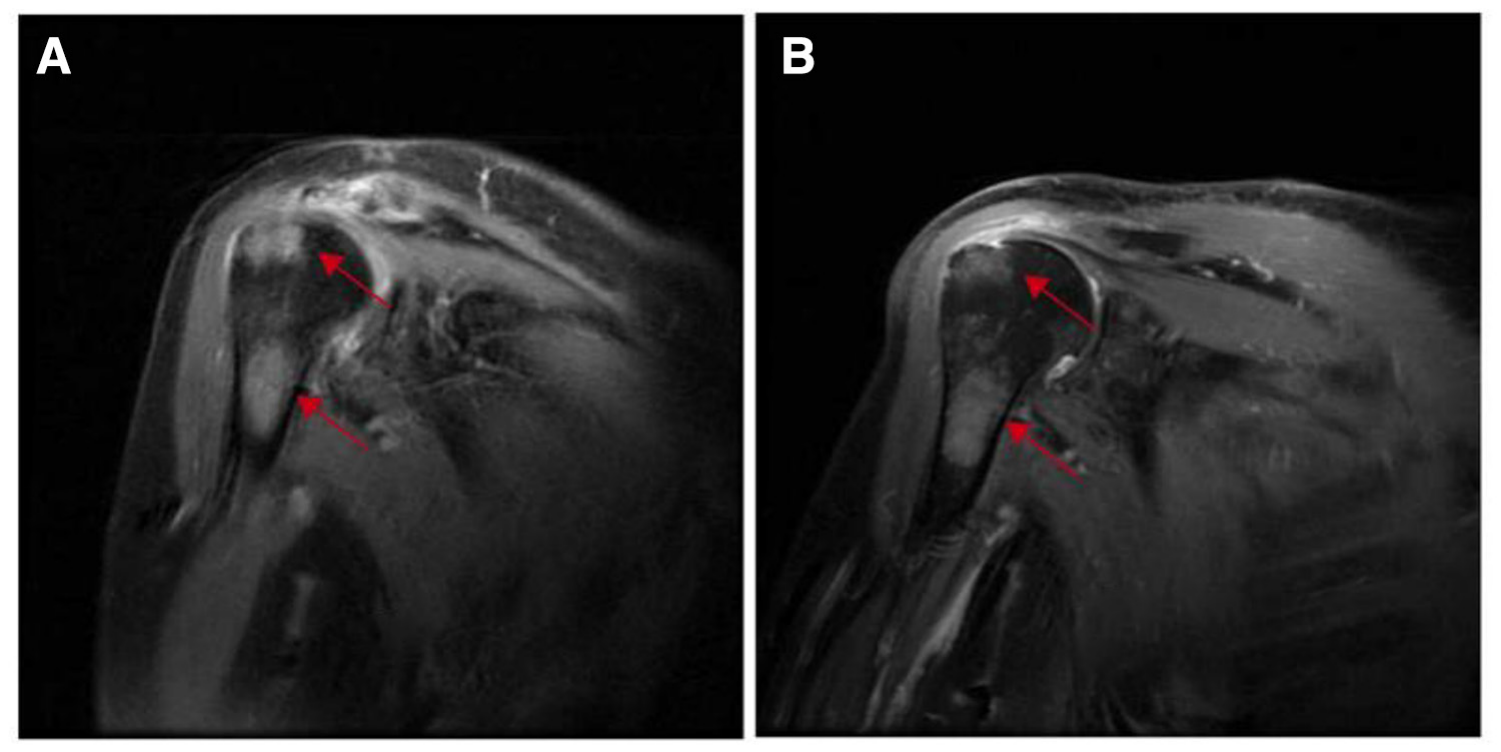
MRI of the right humeral head and neck at 4 months (A) and 6 months (B) after the initiation of antituberculosis therapy. The images show progressive attenuation of the abnormal signal and obvious regression of the lesion over time.

Follow-up MRI examinations of the sacroiliac joints and the right humeral head at 5 and 4 months after the initiation of antituberculosis treatment revealed a marked reduction in lesion size and decreased edema at both sites, indicating effective treatment of the bone tuberculosis (Fig. 2). Serial MRI of the right humeral head and neck at 4 months and 6 months after the initiation of therapy demonstrated progressive attenuation of the abnormal signal and continued regression of the lesion over the extended follow-up period (Fig. 3), providing robust radiological evidence of a sustained therapeutic response.

## 3. Discussion

This diagnosis of isolated primary bone tuberculosis is supported by converging evidence. The initial nonspecific presentation of recurrent fever without bone pain and multifocal skeletal lesions on PET-CT first suggested hematological malignancy, which was excluded by bone-marrow studies. Although the Xpert signal from the sacroiliac biopsy was at a very low level and both mNGS and mycobacterial culture of bone tissue were negative, several factors support a true-positive result: the specimen was obtained from a normally sterile deep skeletal site where contamination is unlikely; osteoarticular tuberculosis is characteristically paucibacillary with limited culture yield, as reflected by the need for 5 sampling procedures to obtain adequate material. The diagnosis is further corroborated by the positive T-SPOT.TB, granulomatous histopathology, the absence of an rpoB resistance mutation, characteristic imaging, and the marked radiological regression after standard antituberculosis therapy. Sarcoidosis, fungal infection, and nontuberculous mycobacterial disease were ruled out. Given the more than 2 years of ruxolitinib therapy, this isolated skeletal tuberculosis is considered secondary to the drug’s immunosuppressive effect.

Tuberculosis is a recognized complication of ruxolitinib therapy.^[15]^ No cases were reported in the initial phase of the JUMP study (1144 patients),^[16]^ likely reflecting the limited sample size and follow-up duration at that stage. As research expanded, tuberculosis was reported in 0.2% of patients in the Phase IIIb JUMP extended cohort (5/2233),^[17]^ rising to 0.5% in a recent observational study (4/892).^[18]^ In a cohort study involving 65 patients treated with ruxolitinib, 2 cases (3%) of mycobacterial infection were observed.^[19]^ In recent years, an increasing number of relevant case reports have gradually emerged.^[14,20,21]^ A literature review found that among 13 patients who developed tuberculosis symptoms while using ruxolitinib, 86% presented with disseminated tuberculosis, with 50% of these cases resulting in death.^[22]^ These figures indicate that the risk of tuberculosis associated with ruxolitinib has become increasingly apparent as exposure accumulates. This requires clinicians to remain highly vigilant.

To place this case in context, we searched PubMed, Embase, Web of Science, and CNKI from database inception to 2026, using the terms “ruxolitinib” combined with “tuberculosis,” “bone tuberculosis,” “osteoarticular tuberculosis,” and “JAK inhibitor.” We included reports of microbiologically or histologically confirmed tuberculosis in patients receiving ruxolitinib and excluded non-ruxolitinib JAK-inhibitor cases, nontuberculous mycobacterial infections, and records without extractable case-level data; the identified cases are summarized in Table 1. Notably, only one of the 17 cases had documented pretreatment tuberculosis screening; the remaining 16, including the present case, had no recorded IGRA result, underscoring a genuine and persistent gap in clinical practice. Hirai et al reached the same conclusion, arguing that latent tuberculosis should be screened for and treated before ruxolitinib is started,^[22]^ a recommendation reinforced by the disseminated and often fatal course seen when infection is detected late. The reported cases encompass disseminated tuberculosis involving the lungs, bones, peritoneum, and lymph nodes^[14,22,23,30]^ miliary tuberculosis manifesting as a neck mass^[24]^ or psoas muscle tuberculosis abscess,^[21]^ all share the common feature of lesions accompanying or secondary to infections in other systems. This finding demonstrates that ruxolitinib-associated tuberculosis can manifest as isolated skeletal disease without systemic dissemination, a pattern not previously described.

**Table 1:** Summary of previously reported cases of ruxolitinib-associated tuberculosis.

Study (ref.)	Age/Sex	Underlying disease	Ruxolitinib duration	TB type and sites	IGRA before ruxolitinib	Ruxolitinib therapy after diagnosis of infection	Outcome
Colomba 2012^[23]^	Not specified/M	MF	2 months	Disseminated (lung, inguinal lymph node)	Not specified	Not specified	Not specified
Shamil 2015^[24]^	78/F	MF	Not specified	Miliary TB presenting as neck mass	Not specified	Stopped	Alive
Panda 2016^[25]^	35/F	MF	2 years	Disseminated (hepatosplenic, mesenteric, renal)	Not specified	6 weeks continued, stopped	Alive
Tsukamoto 2018^[26]^	73/M	PMF	4 months	disseminated (sputum, stomach, blood, urine)	Not specified	Stopped	Died
Lescuyer 2019^[19]^	73/M	PMF	6 months	Pulmonary, Brain	Not specified	Stopped	Dead
Sakiyama 2019^[27]^	76/M	Polycythaemia vera	28 weeks	Tuberculous peritonitis	Not specified	Not specified	Reported
Khalid 2021^[28]^	78/F	Primary thrombocythaemia	3 years	Pulmonary	Not specified	Not specified	Died
Khalid 2021^[28]^	62/M	PMF	13 months	Pulmonary	Not specified	continued	Alive
Khalid 2021^[28]^	54/M	PMF	7 months	Lymph nodes	Not specified	Stopped	Alive
Hirai 2020^[22]^	80/M	PMF	10 months	Disseminated (spondylitis, lung, skin)	Not specified	4 months continued, stopped	Dead
Santoro 2021^[29]^	38/F	Primary thrombocythaemia	2 months	Isolated nodal TB reactivation	positive Mantoux test	Stopped	Alive
Tiwari 2022^[20]^	48/M	PMF	4 months	Pulmonary, Ascitis	Not specified	gradual tapering in 2 weeks, stopped	Alive
Tiwari 2022^[20]^	50/M	PMF	2 months	Lymphadenitis	Not specified	Stopped	Lost
Loutsou 2024^[30]^	70/F	PV/MF	2 years	Pulmonary	Not specified	Stopped	Alive
Chen 2024^[21]^	64/F	PMF	3 years	Psoas muscle TB abscess	Not specified	Stopped	Alive
Gerard 2025^[14]^	80/Not specified	PMF	16 months	Disseminated (peritoneal, lymph node, pleuropulmonary, muscular, multiple bone lesions)	None	Stopped	Alive
Present case	68/F	Primary thrombocythaemia	2 years	Isolated primary bone TB (sacroiliac joints, right humeral head)	Not specified	continued	Alive

F = female, M = male, MF = myelofibrosis, PMF = primary myelofibrosis, PV = polycythemia vera, TB = tuberculosis.

Analysis of the timing of infection onset reveals cases occurring as early as the second month of ruxolitinib treatment,^[23]^ while others, such as the present case, emerged only after more than 2 years of therapy.^[25]^ Across the 17 cases in Table 1, five presented within 4 months, four between six and thirteen months, and the remainder after two or more years. The risk is therefore not confined to the early treatment period but persists with prolonged therapy, consistent with the mechanism of sustained JAK-STAT pathway inhibition progressively impairing T-cell and macrophage function and reducing cytokine output, including IL-6 and TNF-α, thereby diminishing clearance of intracellular *Mycobacterium tuberculosis*. It facilitates a more precise analysis of the relationship between drug dosage, duration of treatment, and the risk of tuberculosis infection. Concurrently, it serves as a reminder to integrate monitoring throughout the entire course of treatment. Particular vigilance is required for patients who have been on medication for over a year, with heightened awareness of tuberculosis infection-related symptoms such as unexplained fever and bone pain, to prevent missed diagnoses due to insufficient monitoring intervals.

The patient’s primary thrombocythaemia required ongoing ruxolitinib. Treatment was not discontinued. Following antituberculosis therapy, bone lesions in the sacroiliac joints and right humeral head have diminished, supporting the view that tuberculosis can be managed without mandatory drug withdrawal in selected patients. In Table 1, two cases continued ruxolitinib after tuberculosis diagnosis and both survived, whereas the 4 fatal cases all involved disseminated tuberculosis in patients aged 73 to 80 years, three of whom had discontinued ruxolitinib. Although causality cannot be established from this small series, the data suggest that the decision to continue or stop ruxolitinib should be individualized according to tuberculosis extent, severity of the underlying myeloproliferative neoplasm, and patient condition, rather than applied uniformly.

Several treatment considerations merit emphasis. Ruxolitinib and rifampicin are both metabolized by CYP3A4. Rifampicin is a potent inducer that markedly reduces ruxolitinib exposure^[31]^ and may compromise disease control. Platelet counts and disease activity should be monitored closely during concurrent use, with dose adjustment as needed; substitution with rifabutin, a weaker inducer, may be considered where appropriate. Before tuberculosis was confirmed, the patient had received empirical levofloxacin for presumed bacterial infection at the referring hospital; fever resolved, but fatigue persisted and prompted further evaluation. In retrospect, defervescence likely reflected the antimycobacterial activity of levofloxacin as a second-line antituberculosis agent rather than resolution of a bacterial infection. This illustrates an important pitfall: fluoroquinolone use can mask tuberculosis symptoms and risk promoting resistance,^[32]^ delaying diagnosis. The standard four-drug regimen was started only after bone tuberculosis was confirmed, at which point empirical antibiotics were discontinued. Similarly, low-dose prednisolone had been given empirically for presumed inflammatory sacroiliitis before the diagnosis was established; corticosteroid use in an already immunosuppressed patient carries risk^[33]^ and was stopped once tuberculosis was confirmed. These points underscore the importance of early multidisciplinary recognition of tuberculosis in patients receiving JAK inhibitors.

## 4. Conclusion

To our knowledge, this is the first reported case of ruxolitinib-associated tuberculosis presenting as an isolated skeletal infection without pulmonary or systemic involvement. Latent tuberculosis should be screened before starting ruxolitinib, and vigilance for extrapulmonary tuberculosis should be maintained throughout therapy, with multidisciplinary evaluation of unexplained bone lesions in JAK-inhibitor users. Empirical fluoroquinolone use before diagnosis may transiently mask the disease and delay recognition. CYP3A4-mediated interactions should be anticipated when rifampicin-based therapy is combined with ruxolitinib.

## Author contributions

**Conceptualization:** Liang-Yan Jin, Ting Li.

**Writing – review & editing:** Liang-Yan Jin, Ping Yu, Bao-Liang Wu.

**Resources:** Fan Huo, Ya-Hui Cui.

**Writing – original draft:** Ting Li.
